# “Change is what can actually make the tough times better”: A patient‐centred patient safety intervention delivered in collaboration with hospital volunteers

**DOI:** 10.1111/hex.12835

**Published:** 2018-10-21

**Authors:** Gemma Louch, Mohammed A. Mohammed, Lesley Hughes, Jane O'Hara

**Affiliations:** ^1^ Bradford Institute for Health Research Bradford Royal Infirmary Bradford UK; ^2^ School of Health Studies University of Bradford Bradford UK; ^3^ Leeds Institute of Medical Education University of Leeds Leeds UK

**Keywords:** evaluation, improvement science, patient feedback, patient involvement, patient safety, volunteers

## Abstract

**Background:**

The PRASE (Patient Reporting and Action for a Safe Environment) intervention provides a way to systematically collect patient feedback to support service improvement. To provide a sustainable mechanism for the PRASE intervention, a 2‐year improvement project explored the potential for hospital volunteers to facilitate the collection of PRASE feedback.

**Objective:**

To explore the implementation of the PRASE intervention delivered in collaboration with hospital volunteers from the perspectives of key stakeholders.

**Design:**

A qualitative case study design was utilized across three acute NHS trusts in the United Kingdom between March 2016 and October 2016. Ward level data (staff interviews; action planning meeting recordings; implementation fidelity information) were analysed taking a pen portrait approach. We also carried out focus groups with hospital volunteers and interviews with voluntary services/patient experience staff, which were analysed thematically.

**Results:**

Whilst most ward staff reported feeling engaged with the intervention, there were discordant views on its use and usefulness. The hospital volunteers were positive about their involvement, and on some wards, worked with staff to produce actions to improve services. The voluntary services/patient experience staff participants emphasised the need for PRASE to sit within an organisations’ wider governance structure.

**Conclusion:**

From the perspective of key stakeholders, hospital volunteers facilitating the collection of PRASE feedback is a feasible means of implementing the PRASE intervention. However, the variability around ward staff being able to use the feedback to make changes to services demonstrates that it is this latter part of the PRASE intervention cycle that is more problematic.

## 1 INTRODUCTION

Improving the safety of health care remains a challenging task, with little evidence to suggest that the NHS is getting safer,^1^ and estimates of adverse events remaining static over the past quarter of a century at approximately 1 in 10 hospital admissions.^2^ Quality improvement has long expounded the importance of measuring what needs to be improved, and audit and feedback (A&F) interventions have proliferated as a means of improving services.^3^ However, the evidence for such interventions is equivocal and focuses upon using clinical outcome data to change individual level clinical performance or practice.^4^ Recently, some authors have described the need for organisations to become more sophisticated in the types of data used for service improvement. For example, Dixon‐Woods and colleagues described how organisations should be problem‐sensing rather than comfort‐seeking when gathering data,^5^ and others have called for the better use of organisational soft intelligence to monitor, and act on, organisational quality and safety performance.^6^

Over the past decade, researchers and policymakers have become increasingly interested in the role of patients and families as sources of feedback to improve services. Patient feedback is arguably a type of soft intelligence,^6^ which goes beyond traditional A&F interventions using clinical outcomes or process data. Healthcare organisations globally have implemented a variety of different mechanisms to gather patient feedback, with some becoming routinely collected, such as the friends and family test within the United Kingdom. However, such routine feedback gathering has not to date specifically been designed to elicit the patient view on the *safety* of their care, despite it being now widely accepted that patients can willingly and meaningfully provide such views to healthcare providers.^7^, ^8^

There has been limited evidence to date in terms of interventions to allow staff to use patient feedback about safety to improve service‐level safety performance.^9^ To address this need, a group of UK researchers developed and tested the Patient Reporting and Action for a Safe Environment (PRASE) intervention. The intervention serves as a theory and evidence‐based approach to systematically collect hospital inpatient feedback about safety, together with a framework for staff to interpret and act on that feedback.^7^, ^9^, ^10^, ^11^, ^12^, ^13^, ^14^, ^15^ Whilst the general design of the PRASE intervention is based on principles of A&F, the authors sought to enhance this process within an improvement cycle of feedback gathering, facilitated action planning and implementation of actions.^9^ Patient feedback about the safety of their care is collected using two measurement tools, (a) the Patient Measure of Safety (PMOS)—a 44‐item theory‐based questionnaire which focuses on nine key domains of safety^10^, ^11^ and (b) the Patient Incident Reporting Tool (PIRT) which elicits detailed safety concerns and/or positive experiences from patients.^7^, ^9^ Following a phase of feedback being collected, an iterative cycle of action planning follows, where staff come together in an Action Planning Meeting (APM) to consider the feedback (in the form of a feedback report) and plan actions to facilitate service improvements. This codesigned intervention was developed and tested between 2010 and 2015.

In order to provide a sustainable mechanism for the PRASE intervention beyond a research study context—a 2‐year improvement project subsequently explored the potential for hospital volunteers to facilitate the collection of feedback within PRASE. The PRASE in collaboration with hospital volunteers project was implemented at three NHS trusts (at three hospital sites), across selected wards. A two‐stage evaluation ran alongside implementation. The results of the formative evaluation which explored the perceptions of key stakeholders throughout the pilot phase have been published previously.^16^ A summative evaluation followed, which coincided with the PRASE intervention being scaled up and spread across additional wards. This evaluation phase focussed on (a) the ward level experience and (b) the ongoing experiences of hospital volunteers and voluntary services/patient experience staff. We aimed to explore the implementation of PRASE in collaboration with hospital volunteers from the perspectives of these key stakeholders, to gain an in‐depth understanding of each ward's PRASE journey, and a collective account of implementation.

### 1.1 Research questions

- What is the ward level experience of the PRASE intervention delivered in collaboration with hospital volunteers?
- What are the on‐going experiences of hospital volunteers, voluntary services and patient experience staff involved in the PRASE intervention?

## 2 METHOD

### 2.1 Patient involvement

A full description of patient involvement in the design and conduct for the wider project has been published previously.^16^ The research aim initially arose from discussions about the sustainability of the PRASE intervention with patients and healthcare professionals. Additionally, a patient representative was part of the initial application for funding, and was invited to attend all steering group meetings.

### 2.2 Design

**Figure 1:**
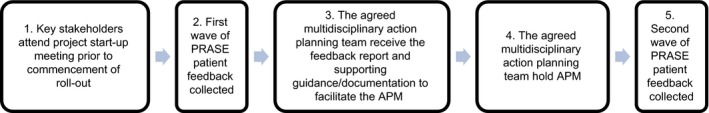
Flow diagram of key PRASE ward activities

Given the relatively small number of participating wards (N = 7), we utilized a case study approach.^17^ This combined different types of data including the following: semi‐structured interviews with ward staff (one‐one and dyad interviews); researcher notes of APM audio recordings; and information pertaining to implementation fidelity. For each ward, we aimed to produce a synthesised account of their PRASE journey. In order to generate a means of assessing implementation fidelity, the key PRASE ward activities were predefined by the implementation team (see Figure 1). These activities were based on a version of the PRASE intervention programme theory agreed by the wider project team at the end of the pilot phase.^16^ Focus groups with hospital volunteers and semi‐structured interviews with voluntary services/patient experience staff were conducted at two time points and focussed on the participants’ experiences of their involvement in PRASE and their views on implementation, with specific questions varying for each participant group.

### 2.3 Setting and sample

Three acute NHS trusts in the United Kingdom were involved in the improvement project, and data collection took place between March 2016 and October 2016. The project was led by a central project team at the lead trust (trust 1). Seven wards were involved in the summative evaluation (roll‐out wards). We refer to each of the wards by pseudonyms (Ward A – Ward G). APMs were digitally recorded for five of the seven wards; two wards did not complete an APM, and eight staff (from four wards) participated in interviews (range 9‐39 minutes; average 26 minutes). We conducted four focus groups with hospital volunteers (n = 13),^A^ and seven interviews with voluntary services/patient experience staff at the beginning and end of the roll‐out phase (n = 5).^B^ More information regarding the data sources, characteristics of ward and study participants and the characteristics of the NHS trusts and services/departments involved in delivering the project are presented in Table 1.

**Table 1:** Data sources, characteristics of the trusts, wards and study participants and services/departments involved at each site^a^

	**Stage**	**Duration**	**Sex, n, (ID codes)**	**Age, mean (SD)**
Trust 1 (Foundation status – Teaching hospital) 900+ bed hospital; Central project team, Voluntary services team				
Hospital volunteers focus groups (FGs)	Early	62 min	F, n = 4; M, n = 2 (V1,V8,V10,V11,V16,V17)	69.67 (3.78)
	End	65 min	F, n = 2; M, n = 3 (V8,V10,V16,V19,V20)	69.00 (4.85)
	**Stage**	**Duration**	**ID codes**	
Voluntary services/patient experience staff interviews	Early	31 min	VS/PE1	
	Early	34 min	VS/PE4	
	End	24 min	VS/PE1	
**Ward level data (included in pen portrait)**	**Pseudonym, Specialty**	**Ward staff interviews (n)**	**APM recording (duration)**	
	A, Care of the Elderly	Y (1: n = 1; 2: n = 2)	Y (72 min)	
	B, Trauma & Orthopaedics	Y (1: n = 1; 2: n = 1)	Y (66 min)	
	C, Cardiology	‐	Y (62 min)	
	**Stage**	**Duration**	**Sex, n, (ID codes)**	**Age, mean (SD)**
Trust 2 (Foundation status) 500+ bed hospital; Central project team, Voluntary services team, Patient experience team, Research and development team				
Hospital volunteers focus groups (FGs)	Early	67 min	F, n = 3 (V13,V15,V18)	59.33 (9.61)
	End	43 min	F, n = 2 (V21,V22)	66.50 (3.54)
	**Stage**	**Duration**	**ID codes**	
Voluntary services/patient experience staff interviews	Early	32 min	VS/PE2	
	End	30 min	VS/PE2	
	End	29 min	VS/PE3	
	End	37 min	VS/PE5	
**Ward level data (included in pen portrait)**	**Pseudonym, Specialty**	**Ward staff interviews (n)**	**APM recording (duration)**	
	D, Paediatrics	Y (1: n = 2)	Y (67 min)	
	E, Trauma & Orthopaedics	Y (1: n = 1)	Y (31 min)	
Trust 3 700+ bed hospital; Central project team, Local improvement team				
Hospital volunteers focus groups (FGs)	‐	‐	‐	‐
Voluntary services/patient experience staff interviews	‐	‐	‐	
**Ward level data (included in pen portrait)**	**Pseudonym, Specialty**	**Ward staff interviews (n)**	**APM recording (duration)**	
	F, General Surgery	‐	‐	
	G, General Surgery	‐	‐	

*Note*: F = Female; M = Male; SD = Standard Deviation; APM = Action Planning Meeting; Y = yes.

a To protect the anonymity of the participants, we have not included the job titles of the voluntary services, patient experience and ward staff.

#### 2.3.1 Evaluation procedure

Hospital volunteer participants were invited to take part in the focus groups by a member of the local project implementation team. Staff participants (ward, voluntary services and patient experience) were approached directly by an evaluation researcher and invited to participate. Participant information sheets were distributed in advance, and written informed consent obtained from all participants. Focus groups and interviews took place in a private room on the hospital sites. The implementation team facilitator recorded ward APMs.^C^ At the start of APMs, the implementation team facilitator confirmed with the participants that they were happy for the meeting to be audio recorded for evaluation purposes.

### 2.4 Data analysis

In an earlier process evaluation of the PRASE intervention, Sheard and colleagues^15^ report how pen portraits have been used in applied health research in qualitative studies to provide a narrative account of a typical participant, or as an analytic aide memoir. Recognising the lack of methodological literature around the construction of a pen portrait, they describe how they “created a basic structure for the pen portraits which centred on the writing of a linear, longitudinal account of how each ward had engaged with relevant key components of the intervention and the contextual factors which influenced this.”^15(p3)^ This previous work guided our approach, and we endeavoured to produce a rich account of the journey of each ward.

The ward staff interviews were digitally recorded and transcribed verbatim, and three researchers listened to the APM recordings and made detailed notes (JOH, LH and GL), with a particular emphasis on the role of hospital volunteers in the ward APMs. An account of implementation fidelity was also produced (see Table 2). We developed a proforma to facilitate the synthesis of these data sources. The proforma aimed to elicit specific information for each ward, for example, timeline of PRASE activities, staff views regarding: hospital volunteer involvement; APMs (eg how actions were decided, role of facilitation); ward involvement in other initiatives. Although the proforma specified a priori information of interest, the approach also allowed for emergent concepts. Three researchers (JOH, LH and GL) synthesised these data sources to produce a pen portrait narrative account of each ward's PRASE journey. A broader level synthesis of the ward pen portraits was generated by two researchers (JOH and GL). An example pen portrait for Ward A is provided in Appendix 1.

**Table 2:** Implementation fidelity in relation to key PRASE ward activities

Activity	Implementation fidelity						
	Ward A	Ward B	Ward C	Ward D	Ward E	Ward F	Ward G
(1) Key stakeholders attend project start‐up meeting prior to commencement of roll‐out	Y	Y	Y	Y	Y	Y	Y
(2) First wave of PRASE patient feedback collected	Y	Y	Y	Y	Y	Y	Y
(3) The agreed multidisciplinary action planning team receive the feedback report and supporting guidance/documentation to facilitate the APM	Y	Y	Y	Y	Y	N	N
(4) The agreed action planning team hold APM	Y	Y	Y	Y	Y	N	N
Multidisciplinary	Y	N	Y	Y	N	‐	‐
Number of attendees	7	5	5	3	2	‐	‐
Hospital volunteer(s) present	Y	Y	Y	N	N	‐	‐
(5) Second wave of PRASE patient feedback collected	Y	Y	Y	N	N	N	N

*Note*: Y = yes; N = no; APM = Action Planning Meeting.

The hospital volunteer focus groups and voluntary services/patient experience staff interviews were digitally recorded and transcribed verbatim. In a series of analysis meetings, two researchers (JOH and GL) took a thematic approach to analysis allowing for both a priori and emergent concepts and themes, with disagreements resolved through discussion.

Subsequently, the same two researchers met in an intense analysis session to generate meta‐themes. The aim of this session was to have a discussion of commonality and differences across the data at a meta, abstract level in order to synthesise the findings from the broader level synthesis of the ward pen portraits and the themes from the hospital volunteer focus groups and voluntary services/patient experience staff interviews.

### 2.5 Ethics and governance

The appropriate governance approvals were sought for each research site, and ethical approval was granted by the University of Bradford, Humanities, Social and Health Sciences Research Ethics Panel.^D^

### 2.6 PRASE intervention

The development and testing of the PRASE intervention has been fully described in previous published work.^7^, ^9^, ^10^, ^11^, ^12^, ^13^, ^14^, ^15^ We present a brief description below:

- patient feedback gathered on:
  - leading indicators of safety (PMOS^10^, ^11^)
  - lagging indicators of safety (PIRT^7^, ^9^)
- patient feedback collated into a feedback report
- report considered within a multidisciplinary action planning team.^E^
- action plans made

The intervention was designed to be cyclical, and time frames are not specified for these activities above what is described in the published literature, which reports 6‐month cycles.^F^

## 3 RESULTS

We now describe the five meta‐level themes. Not all wards progressed through the planned implementation of the intervention and participation in evaluation activities differed at trust and ward level. For instance, volunteer focus groups and voluntary services/patient experience staff interviews were not held at trust 3, and APMs did not take place within the summative evaluation phase at this site. Therefore, themes 1‐4 represent a synthesis of the available data sources, which principally relate to trusts 1 and 2, and theme 5 relates to all trusts.

### 3.1 Legitimacy and validity of PRASE

Whilst most ward staff reported feeling engaged with, and positive about PRASE, there were discordant views on its use and usefulness. Most clinical staff seemed to be engaged in the philosophy behind PRASE. Indeed, for many wards, PRASE was regarded as being superior to other forms of patient feedback about care. The evidence‐base associated with PRASE was viewed as a positive supporting feature.

… Staff were keen to improve the service and wanted to use evidence‐based approaches like PRASE to ensure they are moving in the right direction to improve patient care.

Ward B pen portrait excerpt

However, whilst some staff supported gathering patients’ feedback about care, they were less convinced that PRASE was the best mechanism for achieving this. For such staff, PRASE did not seem to have face validity, that is, understanding its relevance as a safety intervention, and there was confusion around how PMOS items (based on contributory factors) related to ward safety.

….it seemed that the lead nurse was unclear about the overall concept behind PRASE or how the data could be utilised to implement improvement…….

….. *we tried to pick actions out of each issue but the issues were no longer relevant. We don’t know which patient it refers to we don’t have any background on them and giving us these 12 months on – it is too late*.

Ward E pen portrait excerpt

A significant feature of the ward experiences was the degree to which the feedback collected was seen as legitimate, and the impact of this upon engagement. Where ward staff showed strong engagement with PRASE, patient feedback about safety elicited by the measurement tools was regarded as useful. Further, patient feedback was regarded by some ward staff as only valid when timely, or where patients were identifiable, allowing ward staff to act on the feedback in, or close to, real‐time. In one sense, there appeared to be some scepticism about the usefulness of PRASE feedback to support more systemic service improvement over time. This scepticism was further compounded by the view of some ward staff that PRASE simply duplicated other forms of patient feedback (eg friends and family test), which, in the case of one ward in particular, was regarded as more valuable, as it was received more timely.

The volunteers and voluntary services/patient experience staff bought into the philosophy of the PRASE intervention. The volunteers saw value in its cyclical process and recognized the need to keep measuring and action planning. Voluntary services/patient experience staff questioned how PRASE feedback could be combined with other safety data and reflected on the uniqueness of PRASE feedback, with one participant describing it as boundary spanning data. Interestingly, one participant commented that for longevity, PRASE needs to be owned by the ward.

……..*I would see the operational action planning about improvements, probably absolutely needing to be owned by the ward, by the clinical teams, by the wider governance within their clinical business unit*……

Voluntary services/patient experience staff (VS/PE5)

### 3.2 Independence of the volunteer role

Ward staff were almost uniformly positive about the PRASE volunteer role, seeing value in the independent collection of patient feedback. The collective experience of working with the volunteers seemed to have been productive and smooth. However, some ward staff had questions regarding the capability of the volunteers to adequately capture feedback from more challenging patient groups (eg patients with dementia), and the impact of this on the validity of the data, as illustrated below.

…..the staff interviewed felt that at times the patients being asked to provide feedback might have had some level of cognitive impairment which may not have been accounted for in the feedback report. The ward queried whether the ‘Dementia Nurse Specialist’ could be involved in future training for taking feedback from this group, as one staff member commented:

…*you don't want to exclude a whole group of people and you want as much as possible to capture some of the views of people who do have some mild dementia or confusion, so that's quite subtle*…

Ward A pen portrait excerpt

In the three wards where volunteers had participated in an APM (at trust 1), the response to their involvement was positive across all stakeholders. The volunteers had a strong voice at the APMs and often brought a much‐needed positive attitude and on some occasions humour, and contributed to the actions planned. One benefit of their involvement was the added clarity they brought to the patient feedback, translating some of the patient responses, and bringing transparency to the discussion and action plans. Indeed, ward staff commented that they valued the independence of the volunteers and their ability to contextualise information in the report.

….The volunteers were also able to usefully bring some context to the patient feedback, and seemed to encourage a focus on what was important to the patients surveyed.

Ward C pen portrait excerpt

Involving the volunteers in the APMs came about coincidentally and was one of the recommendations arising from the formative evaluation phase.^16^ This additional layer of involvement seemed to serve as a means of closing the loop from the volunteer perspective. In the focus groups, the volunteers who had been involved in these meetings felt their involvement brought an element of public accountability to the action plans. The volunteers and voluntary services/patient experience staff were keen for this additional dimension to the role.

…*because it gives them that reassurance that what they are taking on‐board, we’re taking on‐board and we’re doing something about it and that gives them the confidence to say to people while doing the interview,well this is what we’ve done*…

Voluntary services/patient experience staff (VS/PE3)

…*I have taken [name] back there in, it would be March to show her what the problem was and I asked at the last meeting we had, you know, what have you done? What’s happened?*…

Hospital volunteer, Male (V16)

Involvement in the APMs also provided a useful training opportunity for the volunteers, as discussions highlighted how the feedback they gathered was being interpreted and how they could facilitate patients to expand further.

….*I think it's important because obviously sometimes you can capture things in limited ways and obviously then they want it in detail but one thing that struck me at one of our meetings was about how the data is then interpreted*…..

Hospital volunteer, Female (V10)

### 3.3 Supporting and enriching the experience of volunteers

In the early stage focus groups, volunteers expressed that they were enjoying the role and felt supported. Tailored individual feedback on performance had not yet been received, and requirements for additional support were evident. The need for concrete procedures at the volunteer and organisational level was clear, and from the volunteer perspective, there was some uncertainty around these procedures in the early stages.

…..*What you get is a range of people with different things and probably each person that comes has a different set of expectations of what your manager might be responsible for*….

Hospital volunteer, Female (V13)

In the early stages, the volunteers had many questions about what happens to the feedback collected, and there was a feeling that the loop needed to be closed. In recognition of this, the volunteers were keen to be involved in the APMs, and by the later focus groups, several volunteers had been involved in an APM, which had achieved the intended purpose of “closing the loop” and provided feedback on performance. This additional dimension to the role seems to have led to the volunteers becoming heavily invested in PRASE.

……*it was interesting meeting the action group on the ward with the sister and one of the consultants going through all the reports because it seemed to me that it was more interesting and beneficial to us to do that and see what their reaction was which it was very favourable actually, you know, and there were two or three points that they were going to action and it just made our job if you like more interesting and worthwhile doing that seeing the sort of end result*…

Hospital volunteer, Male (V8)

In earlier interviews, voluntary services/patient experience staff recognized that the spread to additional wards had involved new patient populations and resulted in unanticipated issues. Often, there were too many volunteers for the amount of patients available, and staff reflected that moving forward PRASE recruitment would need to be carefully timed to avoid this saturation. In later interviews, staff emphasised the importance of targeted recruitment, adequate volunteer support and the need to fulfil volunteers’ expectations. They described the upfront and ongoing resources required to implement PRASE with volunteers as sizable, but felt that this investment pays off over the longer term as you would have better skilled, trained, supported and motivated volunteers who want to remain in the process.

[referring to systems in place to support volunteers] *…..It is a huge resource. But it is a worthwhile one you know. I think it is really important but to sustain it I do not know you know it is to me because we are bringing volunteers in, it is part of our job. So you know it is….but it is if it is so driven like this one then the resource is quite a lot the investment really. For our little team. Because there is not many of us so, you know*…..

Voluntary services/patient experience staff (VS/PE1)

Voluntary services/patient experience staff highlighted that the potentially sensitive nature of PRASE feedback meant that escalation of significant patient concerns could intersect multiple teams within an organisation, and issues of patient confidentiality needed further consideration. The need for both soft (volunteer‐level) and hard (organisation‐level) supporting infrastructures was emphasised. For instance, the procedures and guidance in place for volunteers to escalate significant patient concerns need to be fit for purpose from the volunteer perspective, but also an organisation's obligations around escalation and governance.

….*if a project is going to work, it has to become embedded with the existing governance structures*….

Voluntary services/patient experience (VS/PE5)

### 3.4 Challenges of using patient feedback to support service improvement

Ward staff often focussed their efforts on identifying smaller changes that were within their influence, and easier for them to shape the outcome. Such quick fixes were more frequently agreed than systemic solutions. This may have been for a number of reasons. Although perceptions of APM facilitation were often positive, at times, facilitation seemed to encourage a focus on every domain within the report. This may have inadvertently led to a higher volume of actions, at the expense of a more nuanced or systemic approach to change. It is also possible that the lack of multiprofessional approach (grades and disciplines) within some wards perhaps led to a narrower focus or range of solutions. There was an articulated sense of frustration from some staff that PRASE in and of itself cannot supply the resources and support needed to enact real change, and that the intervention, like many improvement efforts, ended up going by the wayside during periods of strain or uncertainty.

There was a general feeling that complex change requiring increased finances or resources were not well supported within the structure of PRASE. Some areas for action were reported as being ‘out of their hands’, this was particularly apparent if other teams and services were required to be engaged, or the action required increased resources such as staffing. One clinician summed up their involvement in PRASE stating:

*It's not easy and when your back is against the wall…. things like these change projects are the things that stop….but it means that change doesn't occur and change is what can actually make the tough times better*…

Ward A pen portrait excerpt

### 3.5 Nature and impact of implementation approach

There were clear differences in ward progress through the implementation activities, and the greatest fidelity was seen at the lead trust (trust 1), and the least fidelity at trust 3. Staff at trust 1 (ward, voluntary services/patient experience) described open and effective communication with the project team. As the core implementation team were embedded within the lead trust, they had undertaken the majority of the early phase pilot work. This may have led to an increased influence to progress the implementation through maximising both existing relationships and the early learning that was specific to their local context. Therefore, establishing a core team locally that has ownership for implementation activity, and that is sensitive to local context, is likely to be important for successful uptake.

Ward staff outside of the lead trust at trust 2 felt there was confusion as to who was responsible for the implementation locally—the site staff or the core project team at the lead trust? Such confusion may have led to reduced effectiveness of the local implementation efforts.

….Staff also reported that the timings associated with the implementation of PRASE on the ward had perhaps not supported a positive experience. As the starting period for PRASE was in October, and the APM did not take place until July of the following year, the ward felt this was too long a gap:

*I felt like I am not on board with it because actually there has been big gaps in between and you sort of forget and then if something else comes up so then feels like you have to refresh yourself again*.

Ward D pen portrait excerpt

This was reinforced from the volunteer and voluntary services/patient experience perspectives. The volunteers felt that at times it was unclear who was leading the project, which impacted on how effectively they were communicated with, speculating that the project may have run smoother if it were led in house. There seemed to be an additional layer of management which blurred lines of responsibility and who the volunteers were accountable to. This was echoed by the experience of voluntary services and patient experience staff at this site, who also described issues around project leadership and lines of responsibility. Such staff also described a disconnect between the fabulous project idea and implementation locally.

*Now this is where the information is coming from. She has said to me they've got enough information on ward X, so I haven't got to go but shouldn't it be my bosses at this end telling me which wards I need to go on or have I got two lots of bosses and I don't know which one?*

Hospital volunteer, Female (V15)

## 4 DISCUSSION

This study explored the experiences of ward staff, hospital volunteers, and those supporting them, in the implementation of a patient‐centred patient safety intervention in collaboration with volunteers. The PRASE intervention had credibility with ward staff, although this was not universal. The legitimacy of patient feedback for service improvement was, however, unanimous across all participants. The role of the hospital volunteers in this intervention was valued by all stakeholders, principally for their independence, and the transparency and accountability that arose through their involvement in APMs. The importance of targeted volunteer recruitment and on‐going support, including feedback on performance, were reinforced. Volunteers were keen to close the loop by attending APMs. We found significant challenges identified in terms of planning and implementing service improvements as part of the PRASE cycle. Finally, we noted differences in the nature of the implementation of the intervention across the three study sites, which may have impacted on the relative success of the intervention. These findings raise a number of interesting issues that we will now discuss further.

The findings relating to the infrastructure, training and support mechanisms required for the implementation of PRASE with hospital volunteers were consistent with the wider literature on the importance of understanding volunteers’ motivations and meeting their expectations for retention.^18^,^19^ Many of these findings reinforced the key themes from the formative evaluation,^16^ and therefore, the discussion reflects more so on novel insights.

Whilst the intervention was received positively by most staff, others were less convinced by its utility and value in supporting safety improvements. It is difficult to decouple this variation from the different implementation approaches. However, we can perhaps be more certain that where PRASE worked best was where relations were good with the volunteers, the feedback was timely, and staff met as a multidisciplinary team to consider the feedback from patients and attempted to make changes using the feedback. Problems arose when these data were not timely, or when there was too much data. The nature of the implementation does seem to impact on the experience of staff involved. The differences between wards in terms of progress through the implementation activities may be due to the implementation team sitting within the lead trust, with implementation activity therefore having a somewhat diluted effectiveness outside of this trust. Further evidence of the key role of implementation is in the need for facilitation of APMs, which mirrors the early developmental work.^9^ Whilst facilitation was regarded positively by staff at the lead trust, there was a less positive perception elsewhere, which perhaps may have been interpreted as undermining the local ownership of the project. These findings speak to issues of complexity, which have been described as “resulting from interactions among many component parts—is a property of both the intervention and the context (or system) into which it is placed.”^20^(p307) There is recognition that defining the key components of an intervention with a view to standardize across sites may not always be the best approach. Indeed, some authors propose standardizing by function, meaning there may be variation in the form components take across sites, but the function a component performs in the local context should be consistent.^21^ Therefore, in our work, it is possible that implementation fidelity may not be an accurate indication that the function of the activities was achieved.

There is increasing attention on how staff use and act on data within health services.^6^, ^14^, ^15^, ^22^ We found an unequivocal lack of movement from data to action within our case study wards, the reasons for which are likely to be manifold and complex. A significant challenge for the implementation of this intervention seemed to be getting health professionals together to discuss, interpret and act on safety‐related data. Such a finding strongly resonates with previous published work describing the PRASE intervention.^9^, ^14^, ^15^ The need to create space and competence for improvement by healthcare staff has been argued as crucial if health services are to improve care and increase efficiency.^23^ With respect to patient feedback specifically, a key challenge for engaging patients in safety or service improvement is using the data as a basis for meaningful change.^24^, ^25^ Indeed, some authors have reported this challenge as a chasm between the activity related to collecting patient feedback, and the complete lack of such feedback filtering down to, and being used by, frontline clinical staff.^26^ Therefore, attempts to mitigate some of the challenges faced by traditional principles of A&F interventions within the design of the PRASE intervention do not appear to have had the desired effect. Two areas are useful to consider when interpreting these findings—the sizeable extant literature on A&F interventions, and the emergent one on how staff use soft intelligence and patient feedback. Whilst the evidence is equivocal for the effectiveness of A&F interventions,^3^, ^4^ this literature has generally focused on changing individual behaviours and practice, with far less known about its use at a service or hospital level. Recent theorising about the mechanisms underpinning A&F interventions may prove useful here. Colquhoun and colleagues^27^ recently presented theory‐informed hypotheses around enhancing the effectiveness of A&F interventions. Whilst there are too many to describe individually, a number of these hypotheses can help us to interpret our findings. First, the most significant condition for effectiveness of A&F is for the feedback to be credible and trustworthy. The feedback from patients was seen as a legitimate source of information for service improvement across our study sample, suggesting a sound basis for the PRASE intervention. This legitimacy may have been further enhanced where volunteers were present within the APMs, which seemed to improve the perceived transparency of the process, and supported further interpretation.

Other conditions for effective A&F interventions are thought to be targeting behaviours that are easy to change, and that the data do not overwhelm and are as simple and specific as possible. Given the almost limitless range of issues identifiable from the combination of PMOS scores and PIRT safety concerns, it is clear that the PRASE feedback report is unlikely to be regarded as simple data, with required changes necessarily within the gift of ward staff. Facilitating the APMs was designed to reduce the potential cognitive load and support directed effort,^9^ but it would appear from our findings that this has not had the desired effect. However, the facilitation role may have been important for a further hypothesis—that A&F interventions are enhanced when they incorporate social discussion about the feedback. A final key hypothesis for enhancing A&F interventions is to optimise the timing of delivery of feedback, which should allow tracking of change over time, be delivered multiple times, and be provided as close as possible to decision making. Given the issues of getting feedback to ward staff in a timely way, thus delaying further PRASE cycles and divorcing the discussion from the context of the feedback when provided, it is unlikely that the implementation of PRASE in our project succeeded in achieving these enhancing conditions.

It is important to note, however, that Colquhoun and colleagues’^27^ proposed enhancing conditions are based on A&F interventions for shaping individual behaviour change. Other work on the use of data (patient feedback, and other forms of soft intelligence) within organisations may provide further insight. Sheard and colleagues^14^ proposed a model—the Patient Feedback Response Framework—for understanding how frontline clinical teams seek to use patient feedback to improve services. They suggest that to meaningfully act on feedback, staff first require normative legitimacy; that is, they regard patient feedback as credible, and gathering patient feedback a moral obligation of health services. Second, staff require structural legitimacy, meaning the autonomy, ownership of, and resources to act on issues arising from patient feedback. Finally, organisational readiness is required in order for staff who believe feedback to be credible, and who feel that issues identified are within their sphere of influence, to achieve more systemic change requiring co‐operation across services, or senior management support. Our findings suggest that whilst most ward staff appeared to have normative legitimacy to act on the feedback, evidence for the second two levels of the framework is more variable, with some staff specifically reporting low levels of organisational readiness to support more sustainable or systemic changes.

One further issue of relevance to our findings is the nature of the PRASE data, and whether it—as soft intelligence—fits within the current dominant improvement approaches within hospitals. Soft intelligence has been described as “the processes and behaviours associated with seeking and interpreting soft data—of the kind that evade easy capture, straightforward classification and simple quantification—to produce forms of knowledge that can provide the basis for intervention.”^6^ ^(p19)^ It has been argued that seeking out data which does not easily conform to measurement is a necessary part of managing patient safety,^28^ even when it is “discomfiting and disruptive.”^6^ ^(p26)^ Our findings seem to resonate with previous suggestions that patient feedback is soft data.^6^, ^22^ PRASE feedback is likely to generate uncertainty rather than certainty in terms of the problems to be solved, requiring further interrogation of extant data or other data gathering. Indeed, within our study, at an organisational level, questions were raised in terms of where PRASE sits within an organisations’ wider governance structure, and the services and departments involved in co‐ordinating its delivery, both of which may facilitate PRASE with volunteers being successfully embedded within an organisation. These issues, combined with the sense of unease patient feedback about their experience of safety may create, perhaps make it less surprising that staff were unable to make anything more than quick fixes based on the report.

## 5 IMPLICATIONS

We present some general recommendations that may support healthcare organisations seeking to implement the PRASE intervention with hospital volunteers:

1. establish a core team locally that has ownership, to ensure implementation is sensitive to the local context to avoid confusion and reduce dilution;
2. ensure the timeliness of patient feedback to ward staff, to increase the legitimacy of the data and support engagement with subsequent action planning;
3. ensure APMs are supported by independent facilitation, which may aid interpretation of the feedback and focuses action planning;
4. invest time in early communications with ward staff, to ensure they have an opportunity to discuss and explore the PRASE hospital volunteer role, the ethos of PRASE, what it adds to the management of patient safety and how it differs from other patient engagement activity;
5. include hospital volunteers in APMs as their involvement may improve interpretation of the feedback, increase transparency of the process and support their ongoing engagement in the intervention.

## 6 LIMITATIONS

The main limitation of this work is that we had limited ability to draw conclusions regarding implementation at trust 3. We were unable to hold volunteer focus groups, with factors such as project progress, volunteer availability and attrition contributing to this. Wards at this site did not hold an APM, and thus, we were unable to interview ward staff. As we could only include in our research the organisations involved in the wider project, this meant we were limited in terms of the number of wards involved, and consequently the number of data sources we could draw upon.

## 7 CONCLUSIONS

Our findings suggest that from the perspectives of key stakeholders, hospital volunteers as conduits of patient feedback about safety are a feasible means of implementing the PRASE intervention. However, the variability and complexity we found in the ability of ward staff to use the feedback to make changes to services demonstrates that it is this latter part of the PRASE intervention cycle that is more problematic.

## CONTRIBUTORS

GL, JOH and MM designed the summative evaluation. GL and JOH conducted the data collection. JOH, LH and GL carried out the analyses. GL and JOH drafted the manuscript, and all authors provided comments and approved the final version.

## COMPETING INTERESTS

None declared.

## NOTES

^A^Several hospital volunteers participated in more than one focus group.

^B^Several voluntary services/patient experience staff only took part in one interview.

^C^The reasoning behind audio recording the action planning meeting was explained (in person or via telephone) to ward managers in advance of the summative evaluation commencing.

^D^Hospital volunteer focus groups, ref: EC1578, May 2014; Voluntary services/patient experience staff interviews, ref: E440, April 2015; Ward staff interviews, ref: E480, September 2015.

^E^Where possible *facilitated* by someone independent of the ward.

^F^Materials associated with the PRASE intervention can be requested online (http://www.improvementacademy.org/tools-and-resources/patient-reporting-and-action-for-a-safe-environment.html).

## APPENDIX 1 Pen portrait Ward A

### Ward A

This ward completed all implementation activities. In terms of overall experience of PRASE, implementation took place against a backdrop of nursing staff shortages, which had affected other improvement initiatives on the ward. However, the staff involved were positive about the PRASE process as collection of the feedback was not disruptive to ward activity, and they felt patients benefitted from the time and interaction with the volunteers. Staff viewed the volunteers as invaluable in terms of the time and independence they brought to collecting feedback. In addition, clinical staff reported finding PRASE data more useful than other sources of patient feedback, such as the friends and family test. The ward highlighted a concern they had about feedback collection with their particular patient group who often have cognitive difficulties. Although they recounted that volunteers always confirmed with senior nursing staff prior to approaching any patients, the staff interviewed felt that at times the patients being asked to provide feedback might have had some level of cognitive impairment which may not have been accounted for in the feedback report. The ward queried whether the “Dementia Nurse Specialist” could be involved in future training for taking feedback from this group, as one staff member commented as follows:
*…you don't want to exclude a whole group of people and you want as much as possible to capture some of the views of people who do have some mild dementia or confusion, so that's quite subtle…*

#### Action planning

The APM was attended by senior nurses, medics and volunteers, although ideally APM participants highlighted that they would have chosen to also involve more junior staff. Volunteers had a strong voice at the APM and staff commented that they valued their clinical independence and ability to contextualise information in the report. The facilitator took the meeting members through the detail of the action plan domain by domain. Nine problems emerged, and 21 individual points to action with 18 of the action points falling to the senior nurses to action. In general, discussion concentrated on negative comments rather than the report as a whole. The majority of actions were simple to implement, but finding spare capacity to fulfil complex actions was more difficult. Focus moved towards the difficulties of improving issues, and there was also concern regarding the need for longer time frames to embed changes due to the staffing shortages:
*It needs to go at their pace because what I don't want them to feel is that there is another thing that's being put onto them when they're already struggling. So, you know, if it takes us longer to make that change than it would normally do then we'll just have to go at that pace*.

Staff appeared to be confident in identifying more immediate “quick fix” actions over which they felt they had influence. Such actions included ordering badges and decaffeinated teabags, although even these were thought to be potentially problematic to implement. The ward was positive about points for action that already had a solution; for example, “poor response to buzzer” was an area for action and the ward already had a new buzzer system in place.

#### Barriers and facilitators

The ward appreciated that a named, skilled, driven facilitator was needed to act as a catalyst for the PRASE process to maintain momentum. After the initial process is established, they felt that the role could be filled by a suitably skilled member of the ward team. There was a general feeling that complex change requiring increased finances or resources were not well supported within the structure of PRASE. Some areas for action were reported as being “out of their hands,” this was particularly apparent if other teams and services were required to be engaged, or the action required increased resources such as staffing. One clinician summed up their involvement in PRASE stating:
*It's not easy and when your back is against the wall…. things like these change projects are the things that stop….but it means that change doesn't occur and change is what can actually make the tough times better*…
